# Production and digital discussion of video presentations by students – a project report

**DOI:** 10.3205/zma001364

**Published:** 2020-12-03

**Authors:** Katja Krug, Johanna Mink

**Affiliations:** 1 Universitätsklinikum Heidelberg, Abteilung Allgemeinmedizin und Versorgungsforschung, Heidelberg, Germany

**Keywords:** e-learning, health professions, interprofessional learning, think pair share, inverted classroom, video presentation

## Abstract

In the summer semester 2020, the module "People in different stages of life" in the Bachelor program "Interprofessional Health Care, B.Sc." had to be adapted to online teaching. The module exam, originally planned as an oral presentation, was presented by the students in interprofessional tandems as a video presentation using case studies. Based on cognitive and social constructivism, all students reflected on these video presentations using the "Think-Pair-Share” method. Similar to regular inverted classroom methods, knowledge transfer through the video presentations was asynchronous and cognitively constructive; accompanying discussion questions of the respective speakers were answered in writing by the fellow students (Think). The students met independently online in small groups on a regular basis to discuss the respective presentations and questions based on personal and professional experiences (Pair; socially constructed). In open online debriefings with the lecturers, the speakers and all interested students, relevant aspects were taken up and discussed again (Share). First experiences showed that the students enjoyed the production of the video presentations and that many of them voluntarily participated in the discussion rounds, in which aspects of health care beyond the presentations were discussed. Considering the experiences made, continuing online teaching in this format may be worthwhile for both lecturers and students.

## Zusammenfassung

Im Rahmen der Umstellung auf Online-Lehre im Sommersemester 2020 wurde im Studiengang „Interprofessionelle Gesundheitsversorgung, B.Sc.“ das Modul „Menschen in verschiedenen Lebensphasen“ entsprechend angepasst. Die ursprünglich als Präsenzreferat geplante Prüfungsleistung wurde von den Student_innen in interprofessionellen Tandems als Videoreferat zu eigenen Fallbeispielen erbracht. Basierend auf kognitivem und sozialem Konstruktivismus wurden diese Videoreferate von allen Student_innen anhand der „Think-Pair-Share-Methode“ reflektiert. Die Wissensvermittlung anhand der Videoreferate fand, ähnlich wie in regulären inverted classroom Methoden, asynchron und kognitiv konstruierend statt; begleitende Diskussionsfragen der jeweiligen Referent_innen wurden von den Kommiliton_innen schriftlich beantwortet (Think). Die Student_innen trafen sich selbstständig online regelmäßig in Kleingruppen, um die jeweiligen Referate und Fragen basierend auf persönlichen und professionellen Erfahrungen zu diskutieren (Pair; sozial konstruiert). In einer offenen Online-Nachbesprechung mit den Dozentinnen, den Referent_innen und allen interessierten Student_innen wurden relevante Aspekte nochmals aufgegriffen und diskutiert (Share). Erste Erfahrungen zeigen, dass die Student_innen Freude an der Produktion der Videoreferate hatten und viele freiwillig an den Diskussionsrunden teilnahmen, in denen über die Referate hinausgehende Aspekte der Gesundheitsversorgung diskutiert wurden. Unter Berücksichtigung der gemachten Erfahrungen kann die Verstetigung dieses Formats gewinnbringend für Dozent_innen und Student_innen sein.

## Introduction

Learning with digital media needs to be embedded in social interaction [1], [2], since knowledge is not only subjectively constructed, but also adjusted and consolidated in social exchange. In interprofessional teaching, this so-called social constructivism is important for the acquisition of competencies in addition to the individual examination of new content in terms of cognitive constructivism [3]. The integration of different forms of learning, such as blended learning and inverted classroom methods, can have positive effects on gaining knowledge and acquiring skills in the training of health care professions [4], [5]. For example, knowledge can be cognitively constructed with the help of videos [6] in an asynchronous format and socially constructed in interactive phases of attendance.

By switching to sole online teaching, the 2-semester module "People in different stages of life" of the Bachelor program "Interprofessional Health Care, B.Sc." was also adapted.

The challenge was to enable independent and collective online learning in a way that supported all students in cognitive and social knowledge construction. In the online format, the presentation was retained as a form of examination and implemented as a video presentation. The production of learning videos enables the independent construction of knowledge since content must be understood and didactically prepared [7]. For reflection and discussion of the video presentations, the "Think-Pair-Share” method [8] was applied online in a modified form, since this method enables the individual, cognitive (Think) as well as the collective, social (Pair, Share) construction of knowledge.

## Module description

### Regular module schedule

The module begins regularly in the 7^th^ semester (winter) providing students with theoretical basic knowledge about health and disease aspects in different stages of life. In the following summer semester, the students usually prepare a presentation in an interprofessional tandem, analysing a case study using a stage theory model [9], [10], [11], identifying opportunities and challenges associated with the stage of life, and analysing interprofessional collaboration. This is based on content from the winter semester, own experiences and profession-specific expertise. The presentations conclude with discussion questions to encourage exchange among fellow students from different health care professions. In this way, the students gain insights into the fields of activity of the other professions and can further develop their own understanding of their roles.

#### Updated module schedule for the summer semester 2020 – online

After the winter semester had taken place regularly, the 16 students in the 8^th^ semester (professional trainees in speech language therapy, physiotherapy, medical radiology assistance, medical laboratory assistance, nursing, orthoptics) received sample videos and a written summary with information on video production at the beginning of the summer semester via an e-learning platform (Moodle); they evaluated the videos and derived criteria for effective videos in individual work. The submitted criteria were summarized by the lecturer in a document and made available to all students via Moodle, based on the constructivist approach.

**Figure 1:**
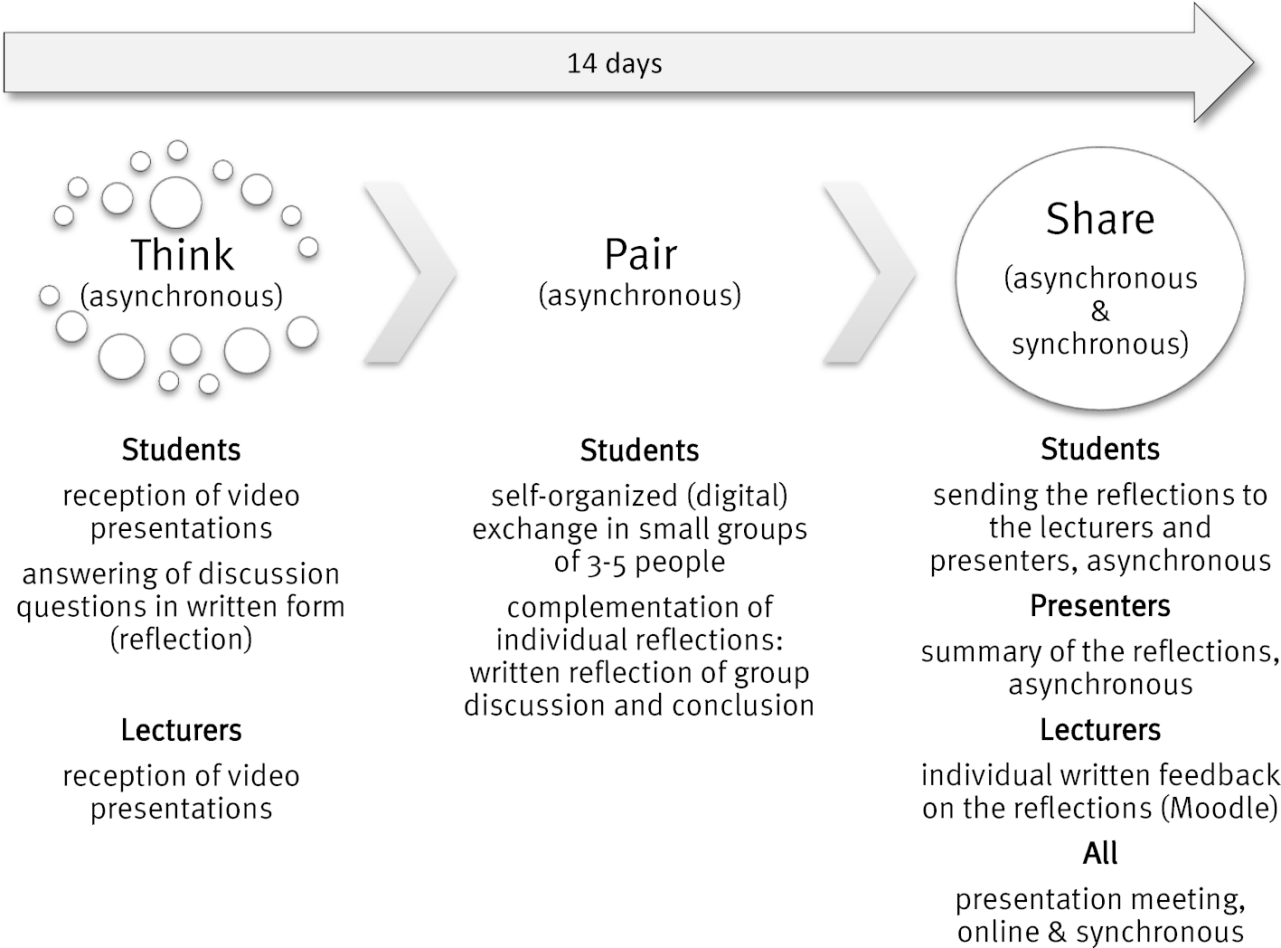
Process of the online “Think-Pair-Share” method; this 14-days cycle was repeated five times in the summer semester.

The students (presenters) created video presentations with concluding discussion questions in interprofessional tandems following the regular requirements of the assignment. A maximum of 2 video presentations were made available to the students via Moodle on 5 fixed dates and were reflected upon and discussed in 14-day cycles using the "Think-Pair-Share” method (see figure 1 (Fig. 1)). In this way, the students were able to access existing knowledge in their own learning time and link it with new knowledge so that the opinions and expertise of all could be integrated.

## Experiences

At present, no structured evaluation is available, only first experiences based on informal feedback from students and lessons learned from the lecturers are described. The students said they enjoyed the production of the video presentations (especially laying techniques and screencasts). The written reflections showed that the students dealt intensively with the case studies and the module content in terms of cognitive knowledge construction (Think). The small group meetings (Pair) promoted independent learning and gave freedom of planning. At least 60% of the students took part in each of the 5 presentation meetings (Share). Topics that went beyond the presentation were discussed, expertise and perspectives were exchanged and social knowledge construction was achieved. The individual feedback of the lecturers, which often provided food for thought for the discussion, was experienced positively by the students as appreciation of their own workload, but was rarely taken up by them in the presentation meeting.

## Discussion

The online application of the "Think-Pair-Share" method has been successful. Video presentations can be retained as an exam format. The production and reception of videos as a substitute for live presentations allows for an individual organisation of learning time and an experience-based occupation with the topic. Structured reflection and discussion enable collaborative and interprofessional learning.

The presentation meetings should preferably be conducted face-to-face to foster blended learning, since online conferences allow less intensive interaction.

For the feedback on the individual reflections, peer feedback could be useful in the context of Think-Pair-Share to promote further social knowledge construction. All in all, a clear formulation of the work assignment for the presentations and discussion groups is essential.

## Competing interests

The authors declare that they have no competing interests.
